# Large Language Models in Spine Surgery: A Scoping Review of Clinical Efficacy, Technical Integration, and Ethical Paradigms

**DOI:** 10.1177/21925682261481450

**Published:** 2026-08-20

**Authors:** Samer G. Salman, Rohan A. Phadke, Anne E. Tatooles, Adithya Nair, Alireza Tavakkoli, James Rizkalla

**Affiliations:** 1 School of Medicine, 3989 Baylor College of Medicine, Houston, TX, United States; 2 Joseph Barnhart Department of Orthopedic Surgery, Baylor College of Medicine, Houston, TX, United States; 3 Department of Computer Science, Human-Machine Perception Laboratory, 6851 University of Nevada, Reno, NV, United States; 4 Department of Orthopaedic Surgery, 22683 Baylor University Medical Center, Dallas, TX, United States

**Keywords:** large language models, spine surgery, artificial intelligence, clinical decision support, patient education, radiology report simplification, retrieval-augmented generation, implementation science

## Abstract

**Study Design:**

Scoping review.

**Objectives:**

To map spine literature on large language models, characterize reported use cases, and identify evidence gaps limiting implementation.

**Methods:**

A scoping review was conducted according to Joanna Briggs Institute methodology and PRISMA-ScR guidance. PubMed, Embase, Scopus, Web of Science, and Cochrane were searched for English-language, peer-reviewed studies published from January 2023 through May 2026 that evaluated large language models in spinal disease, spine surgery, or spine-related care. Eligible studies were synthesized across clinical decision support, triage, patient communication, automation, surgical education, and implementation barriers.

**Results:**

Fifteen studies met inclusion criteria. Most evidence involved early evaluation of commercially available or general-purpose models rather than prospectively validated spine-specific systems. Reported applications included patient education, report simplification, coding support, emergency consultation simulation, spinal cord stimulation referral screening, conservative triage, and surgical education. Performance was strongest for structured text-based tasks, patient communication, documentation support, and simplified decision pathways. Performance was weaker for image interpretation, quantitative radiographic assessment, individualized operative planning, and granular procedure selection. Recurrent limitations included hallucinated or unsupported outputs, unreliable citation generation, limited multimodal capability, privacy and data-governance concerns, bias, unclear medicolegal accountability, and minimal validation.

**Conclusions:**

Large language models are an adjunct in spine surgery, with the near-term role in clinician-supervised, text-centered workflows including patient communication, education, documentation, coding, guideline retrieval, and preliminary triage. Current evidence does not support autonomous diagnostic, radiographic, or operative decision-making. Future studies should prioritize spine-specific retrieval-augmented systems, validated multimodal workflows, privacy-preserving deployment, fairness assessment, and prospective evaluation using clinically meaningful outcomes.

## Introduction

Large language models (LLMs) are increasingly being evaluated across medicine for tasks involving text generation, information retrieval, clinical summarization, patient communication, and decision support. In spine surgery, their potential appeal is clear: clinical care depends on synthesizing complex symptoms, neurologic findings, imaging reports, operative indications, risk profiles, and patient preferences. Because much of this information is documented in narrative or semi-structured form, LLMs may be well suited for tasks that require language-based reasoning, documentation support, or communication between clinicians and patients.^1,2^

Despite this promise, the clinical role of LLMs in spine surgery remains uncertain. Strong performance on general medical benchmarks does not necessarily translate into safe or accurate surgical decision-making. Spine surgery involves high-stakes judgments, including diagnostic localization, operative candidacy, procedural selection, complication risk counseling, and recognition of urgent neurologic conditions.^3,4^ Errors in these settings may have major consequences, particularly if model outputs are inaccurate, incomplete, biased, or presented with unjustified confidence.^1,2,5^ Hallucinated citations, unsupported recommendations, and inconsistent responses across prompts remain important barriers to clinical use.

The existing literature is also heterogeneous. Studies have evaluated LLMs for patient education, board-style examination performance, emergency consultation, procedural coding, diagnostic support, triage, and surgical decision-making, often using different models, prompts, reference standards, and outcome measures. As a result, it remains difficult to determine where LLMs are most reliable, where they remain unsafe, and what types of validation are needed before clinical implementation. This distinction is especially important in spine surgery, where text-only tasks may be fundamentally different from tasks requiring imaging interpretation, procedural judgment, or patient-specific risk assessment.^5-7^

This scoping review maps the current literature on LLM applications in spine surgery from 2023 through 2026. We summarize evidence across clinical decision support, diagnostics and triage, patient education, administrative automation, and medical education, with emphasis on reported performance, methodological limitations, safety concerns, and implementation barriers.

## Methods

This scoping review was conducted in accordance with the Joanna Briggs Institute methodology for scoping reviews and reported according to the Preferred Reporting Items for Systematic Reviews and Meta-Analyses extension for Scoping Reviews guidelines (PRISMA-ScR).^8,9^ A scoping design was selected because large language model research in spine surgery remains early, heterogeneous, and rapidly evolving, making evidence mapping more appropriate than a narrowly focused effectiveness review.

### Search Strategy and Information Sources

A comprehensive literature search was performed in PubMed/MEDLINE, Embase, Scopus, Web of Science, and the Cochrane Library for studies published from January 1, 2023, through May 15, 2026. Free-text search terms were applied to title and abstract fields and combined two core concepts with the Boolean operator AND: (1) large language models and generative artificial intelligence, including “large language model,” “generative artificial intelligence,” “ChatGPT,” “GPT-3,” “GPT-3.5,” “GPT-4,” “GPT-4o,” “GPT-5,” “Gemini,” “Bard,” “Claude,” “Llama,” “Med-PaLM,” and “Microsoft Copilot”; and (2) spine surgery, spinal disease, and spine-specific clinical care, including “spine surgery,” “spinal disease,” “cervical spine,” “thoracic spine,” “lumbar spine,” “spinal fusion,” “spinal cord stimulation,” “scoliosis,” “spinal stenosis,” “disc herniation,” “degenerative disc disease,” “cervical myelopathy,” and “cauda equina.” Broad orthopaedic or neurosurgical terms and task-specific terms such as coding, examination performance, patient education, or triage were not required in the final search, because eligible applications could span multiple clinical and technical domains and were captured through the spine concept. Reference lists of included articles were reviewed to identify additional eligible studies. The complete electronic search strategies, including all search terms, Boolean operators, field restrictions, and applied limits, are provided in the Supplementary Methods.

### Eligibility Criteria

Studies were eligible if they evaluated an LLM or generative AI system in the context of spinal disease, spine surgery, or spine-related clinical care. Eligible designs included randomized trials, cohort studies, cross-sectional evaluations, diagnostic or performance benchmarking studies, and relevant narrative, systematic, or scoping reviews. Publications were limited to English-language, peer-reviewed articles.

Studies were excluded if they focused only on traditional machine-learning models without a language-model interface, addressed general orthopedics or neurosurgery without a dedicated spine component, evaluated AI only for nonclinical tasks, or were conference abstracts, case reports, animal studies, unpublished preprints, or non-peer-reviewed reports.

Secondary literature was included because the objective of this review was to map the scope, distribution, and maturity of large language model applications in spine care rather than to estimate pooled effects. Narrative, systematic, and scoping reviews were the principal available source of information on implementation barriers, governance frameworks, and technical architecture, domains in which primary spine-specific studies remain sparse, and their inclusion is consistent with Joanna Briggs Institute guidance permitting any evidence source relevant to the mapping question.^8^ To avoid duplicate reporting of the same evidence, findings available from both a primary study and a review were counted once and attributed to the primary study, and only the primary study was charted as an included record. Quantitative findings available only from a review are cited to that review explicitly.

### Study Selection and Data Charting

After duplicate removal, titles and abstracts were screened independently and in duplicate by two reviewers, with each reviewer blinded to the other’s decisions. Full-text review was performed by the same two reviewers, again independently. Disagreements at either stage were referred to a third reviewer, who adjudicated the final inclusion decision. A formal inter-reviewer agreement statistic, such as Cohen’s kappa, was not calculated; neither the Joanna Briggs Institute methodology nor the PRISMA-ScR checklist requires a quantitative agreement measure for scoping reviews, and disagreements were infrequent and resolved by third-reviewer adjudication. Data extraction was performed independently by two reviewers using a standardized charting form, with discrepancies reconciled by the third reviewer. Extracted variables included study design, clinical context, LLM model and version, task type, input modality, comparator, outcome measures, key performance findings, implementation setting, and reported limitations.

### Evidence Synthesis

Findings were synthesized descriptively and organized by functional domain: clinical decision support and triage, preoperative modeling and diagnosis, patient-facing communication, administrative automation, and surgical education. Quantitative outcomes, including accuracy, sensitivity, specificity, F1 score, Cohen kappa, and user-rated measures, were tabulated when available. Because the objective was to map the scope and characteristics of the literature rather than estimate pooled treatment effects, meta-analysis was not performed. Formal risk-of-bias appraisal was not conducted, consistent with the exploratory purpose of a scoping review, although methodological limitations of the included evidence were summarized narratively. Model names are reported using a consistent convention: OpenAI models are identified by version, such as GPT-3.5, GPT-4, GPT-4o, and GPT-5, with ChatGPT retained only where a source study evaluated a named consumer product tier, such as ChatGPT-5 Pro, or did not specify the underlying model version; Google models are identified as Bard for the original product and as Gemini with the version number thereafter; Anthropic models are identified as Claude with the version number; and where a source study did not report a model version, the model is described as that study reported it.

## Results

**Figure 1.**
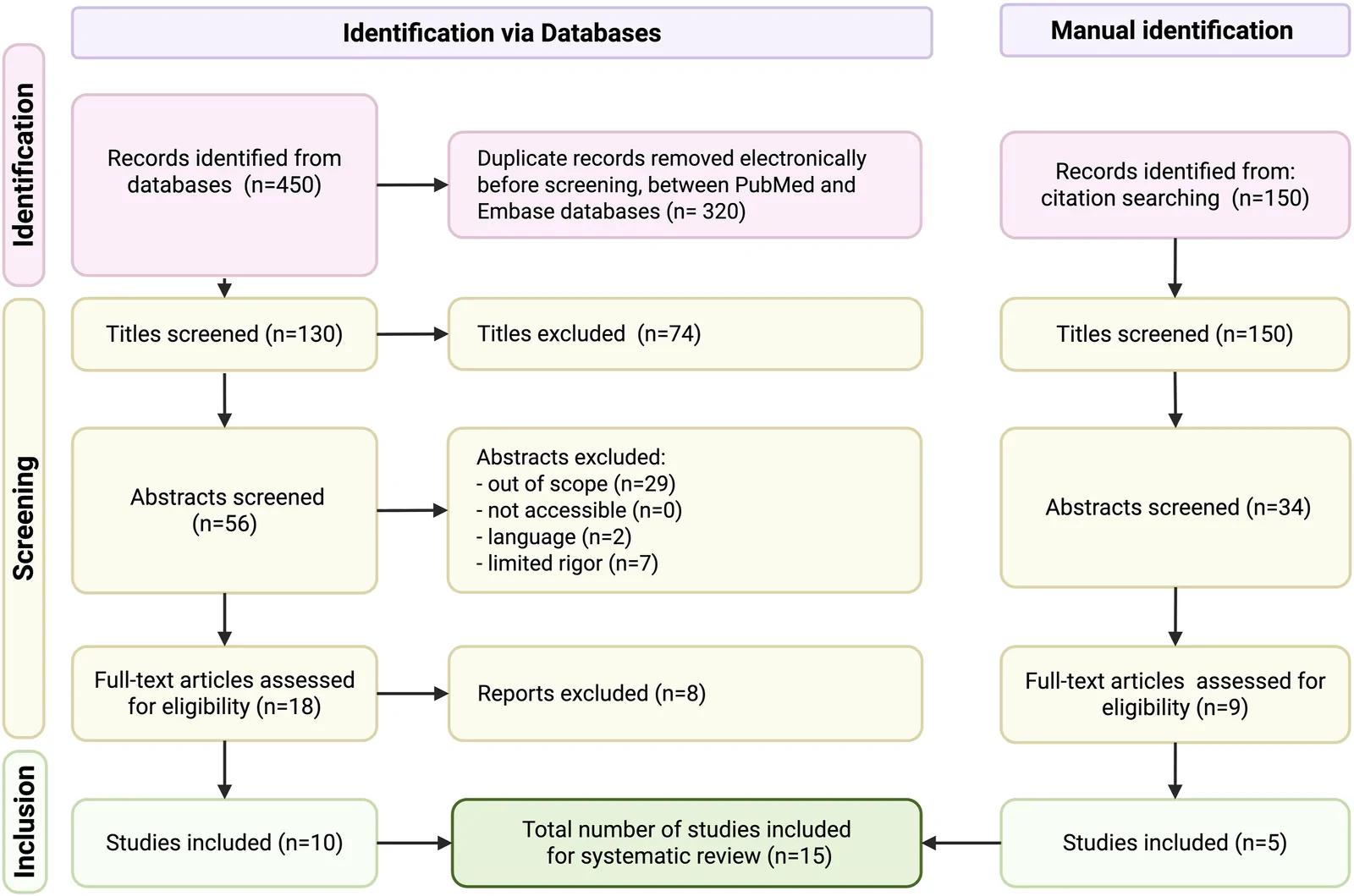
PRISMA-ScR Flow Diagram of Study Identification and Selection. Records were identified through database searching and manual citation searching. After duplicate removal and sequential title, abstract, and full-text screening, 15 studies were included in the scoping review, including 10 from database searches and 5 from manual citation searching. PRISMA-ScR indicates Preferred Reporting Items for Systematic Reviews and Meta-Analyses Extension for Scoping Reviews

**Figure 2.**
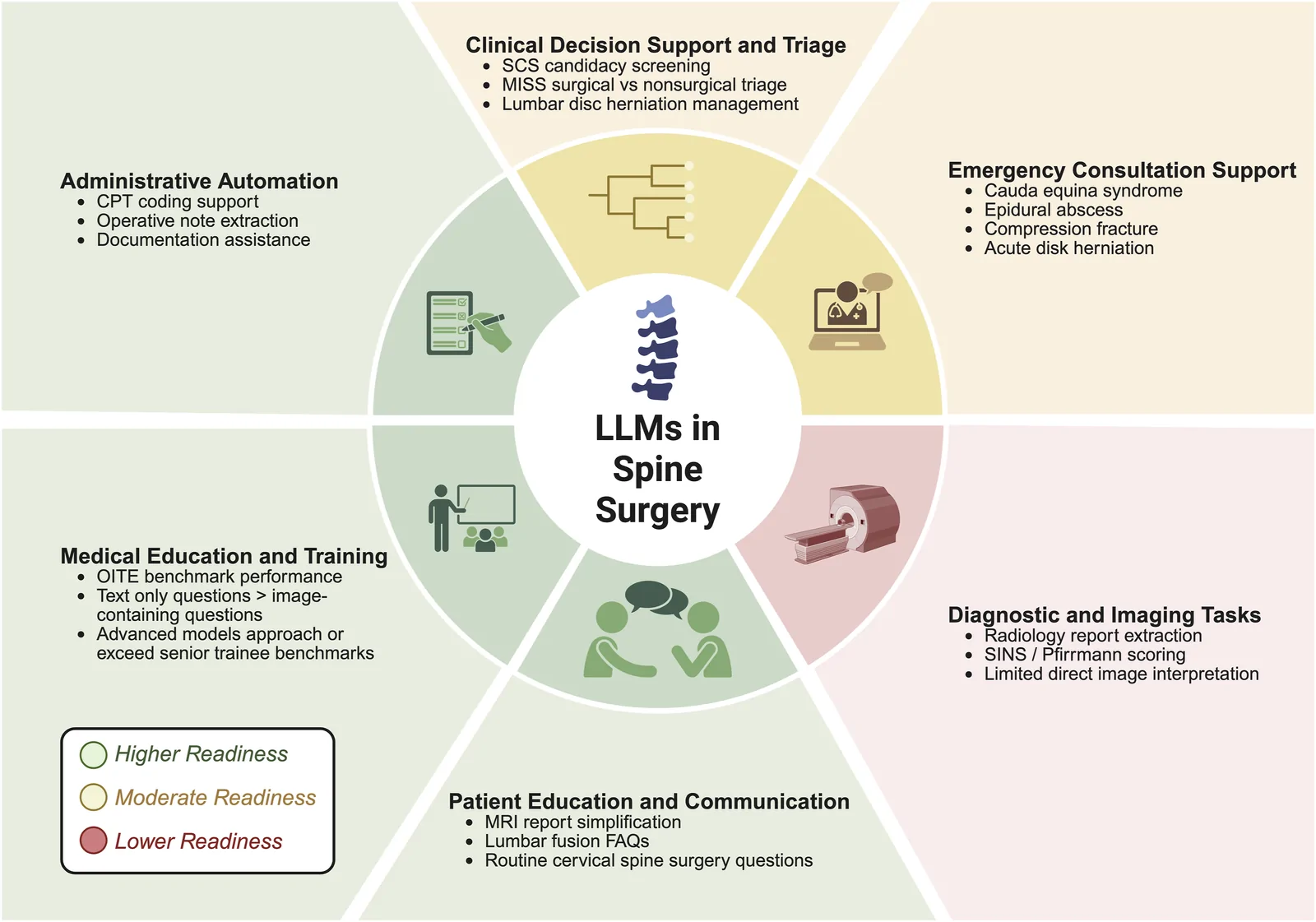
Evidence Map of LLM Applications in Spine Surgery by Functional Domain and Implementation Readiness. Large language model applications in spine surgery were grouped into six functional domains: administrative automation, medical education and training, patient education and communication, clinical decision support and triage, emergency consultation support, and diagnostic and imaging tasks. Relative readiness was highest for structured text-based, documentation, and communication tasks; intermediate for triage, education, and consultation support; and lowest for image-dependent diagnostic tasks or decisions requiring definitive procedural judgment. Readiness categories reflect qualitative synthesis of current evidence and clinical implementation risk. Abbreviations: CPT, Current Procedural Terminology; LLM, large language model; MISS, minimally invasive spine surgery; MRI, magnetic resonance imaging; OITE, Orthopaedic In-Training Examination; SCS, spinal cord stimulation; SINS, Spinal Instability Neoplastic Score

Study selection is summarized in Figure 1. The search identified 450 database records and 150 additional records through citation searching. After electronic duplicate removal, 130 database-derived titles and 150 manually identified titles were screened. Following title, abstract, and full-text review, 15 studies met inclusion criteria, including 10 identified through database searching and 5 through manual citation searching. The literature published from 2023 through 2026 shows rapid expansion of LLM research in spine surgery following the late-2022 release of consumer-facing generative AI tools. Early studies primarily evaluated general medical knowledge and response quality using GPT-3.5, whereas more recent studies examined advanced models, including GPT-5, Gemini 2.5 Pro, GPT-4o, Claude 3.5, and multimodal systems, across progressively more complex spine-specific tasks.^1,10,11^ The included applications clustered into six functional domains, with greatest near-term readiness for structured text-based, communication, education, and administrative tasks and lower readiness for direct imaging interpretation and procedure-specific decision-making (Figure 2).

### Clinical Decision Support and Surgical Triage

LLMs were most consistently evaluated as adjuncts for triage, consultation support, and preliminary decision-making. In the SPINE study, GPT-4 was compared with an expert multidisciplinary team and a rule-based e-Health tool for spinal cord stimulation candidacy among 93 consecutive adults.^12^ The multidisciplinary team recommended spinal cord stimulation for 91.4% of patients, compared with 46.2% for GPT-4 and 54.8% for the e-Health tool.^12^ Agreement between GPT-4 and the multidisciplinary reference standard was fair, with κ = 0.29, indicating that GPT-4 behaved as a conservative, high-specificity screening tool rather than a replacement for expert review.^12^

In minimally invasive spine surgery, procedural decision-making remained more difficult. Across 90 case-report vignettes, agreement with expert-defined procedural categories was slight for ChatGPT-5 Pro and fair for Gemini 2.5 Pro, with κ values of 0.146 and 0.245, respectively.^11^ When the task was simplified to binary surgical versus nonsurgical triage, agreement improved to κ = 0.415 for ChatGPT-5 Pro and κ = 0.587 for Gemini 2.5 Pro.^11^ These findings suggest that current LLMs perform better when identifying broad treatment direction than when selecting specific operative strategies, such as decompression alone versus decompression with fusion.^11^

For lumbar disc herniation, model performance improved when radiology report text was supplemented with clinical information.^13^ GPT-5 achieved 75% accuracy using radiology report text alone, with sensitivity of 0.92 and specificity of 0.58.^13^ After manually summarized clinical information was added, accuracy increased to 85%, with corresponding gains in specificity, positive predictive value, negative predictive value, and F1 score.^13^ Table 1 summarizes reported performance metrics for LLM-based triage, diagnostic support, and consultation tasks.

**Table 1.** Performance Metrics of LLMs in Triage and Decision Support

Clinical application	Year	Country	Study design	Sample size	Task description	Model(s)	Primary result	Key metric	Source
ED Consultation	2025	USA	Comparative simulation study, blinded raters	7 simulated cases (21 questions); 7 spine surgeons; 3 raters	Acute Spine Diagnosis	GPT-4o	Superior to surgeon across 5 metrics	p < 0.05; inter-rater κ = 0.76	14
MISS Triage	2025	USA	Vignette-based cross-sectional study	90 clinical vignettes	Procedural Selection (10 categories)	ChatGPT-5 Pro; Gemini 2.5 Pro	Case-level agreement vs reference	ChatGPT-5 Pro κ = 0.146 (Slight); Gemini 2.5 Pro κ = 0.245 (Fair)	11
MISS Triage	2025	USA	Vignette-based cross-sectional study	90 clinical vignettes	Binary (Surg vs. Non-Surg)	ChatGPT-5 Pro; Gemini 2.5 Pro	Binary triage agreement vs reference	ChatGPT-5 Pro κ = 0.415 (Moderate); Gemini 2.5 Pro κ = 0.587 (Moderate)	11
SCS Triage	2026	Italy	Single-center retrospective cohort study	93 consecutive adults	Candidate Selection vs. MDT	GPT-4	46.2% recommended (vs MDT 91.4%)	κ = 0.29 (Fair)	12
LDH Management	2026	China	Exploratory multicenter retrospective study	48 cases (3 centers)	Surg vs. Conservative	GPT-5	75% accuracy from radiology report text alone; 85% after adding clinical information	F1 score improvement	13

Studies are ordered chronologically by date of first online publication. Country reflects the lead or corresponding institution and the source of the study population; the author groups for^11^ and^12^ were multinational. Abbreviations: ED, emergency department; GPT, generative pretrained transformer; κ, Cohen’s kappa coefficient; LDH, lumbar disc herniation; LLM, large language model; MDT, multidisciplinary team; MISS, minimally invasive spine surgery; Non-Surg, nonsurgical management; SCS, spinal cord stimulation; Surg, surgical management; vs, versus. F1 score represents the harmonic mean of precision and recall. p-values indicate statistical significance testing.

### Acute Spine Emergencies and Consultation Support

LLMs also showed potential in simulated emergency spine consultation tasks. In one comparative evaluation, GPT-4o responses to simulated emergency department consultations were rated higher than responses from board-certified spine surgeons across clinical accuracy, management appropriateness, completeness, helpfulness, and overall quality, with all comparisons reaching statistical significance.^14^ The simulated cases included cauda equina syndrome, epidural abscess, compression fracture, acute lumbar disc herniation, incomplete spinal cord injury, surgical wound drainage, and metastatic spine disease.^14^ Inter-rater agreement was substantial, with κ = 0.76.1^14^ These findings support possible use as a consultation adjunct in time-sensitive settings, although the evidence remains simulation-based rather than prospective clinical validation.

### Diagnostics and Imaging Assessment

Performance was less consistent for imaging-based and quantitative diagnostic tasks. A narrative review through February 2026 reported stronger LLM performance on technical text tasks than on direct image interpretation or quantitative spine measurements.5^5^ In a cross-sectional analysis of 122 scoliosis radiographs, four general-purpose LLMs exceeded clinically accepted Cobb-angle tolerance, and none correctly identified S-shaped scoliosis cases.^15^

In contrast, LLMs performed better when extracting structured information from radiology reports. In 124 spinal-metastasis radiology reports, Claude 3.5 calculated the Spinal Instability Neoplastic Score with an intraclass correlation coefficient of 0.984 compared with an expert reference standard.^16^ GPT-4 has also been reported to extract Pfirrmann grades for lumbar disc degeneration from MRI reports with high agreement.^7^ Overall, the evidence indicates stronger reliability for report-based extraction and scoring than for direct image interpretation.^5,10,11^

### Patient Education and Communication

Patient-facing communication was one of the most consistently favorable application areas. Thoracolumbar MRI reports were typically written at an 11th-to 12th-grade level, whereas GPT-4o-generated explanations reduced reading level to approximately 10th to 11th grade and improved Flesch Reading Ease scores from 32.2 to 53.9, p < 0.001.^17^ Spine surgeons rated these explanations as helpful in nearly all cases.^17^ Other studies reported improved patient understanding after LLM simplification of spine imaging reports, including increases from 6.56 to 8.50 on a 10-point understanding scale, p < 0.001.^7^

LLMs also generated generally satisfactory answers to common spine-procedure questions. For lumbar fusion frequently asked questions, GPT-3.5 and Bard produced satisfactory responses in 97% of cases.^18^ For routine anterior cervical discectomy and fusion questions, LLM responses were rated clearer than physician responses, 75.0% versus 62.5%, p = 0.012, and similar in completeness, 63.0% versus 64.5%, p = 0.404, despite being substantially shorter, 30.0 versus 153.7 words, p < 0.001.^19^ Indirect evidence from a broader patient-forum study also found higher quality and empathy ratings for GPT-3.5 responses than physician responses, although this study was not spine-specific.^20^

However, patient-facing use remained limited by accuracy and source reliability. Prior work reported citation fabrication rates as high as 88.3% in direct patient-facing question-answering contexts.^7^ Models also performed less reliably when asked individualized questions about surgical risks, prognosis, and expected success rates.^18^

### Medical Education and Surgical Training

LLMs showed substantial gains on standardized orthopaedic knowledge benchmarks. In a 412-question Orthopaedic In-Training Examination-style dataset from the 2023 to 2024 cycle, Gemini 2.5 Pro achieved 81.1% accuracy and GPT-5 achieved 76.0% accuracy.^10^ Both exceeded published PGY-5 resident benchmark ranges of 74.2% to 75.3%.^10,21^ In the spine subspecialty category, Gemini 2.5 Pro achieved 84.2% accuracy, compared with 73.7% for GPT-5.^10^

Performance declined on image-containing questions compared with text-only questions. GPT-5 decreased from 79.8% accuracy on text-only questions to 71.6% on image-containing questions, while Gemini 2.5 Pro decreased from 87.2% to 74.2%.^10^ Error analysis showed that GPT-5 errors were most often attributed to faulty clinical reasoning, accounting for 52.5% of errors, whereas Gemini 2.5 Pro errors were most commonly attributed to stem misinterpretation, accounting for 43.6% of errors.^10^ Table 2 summarizes model performance across OITE question categories, resident benchmarks, and dominant error modes.

**Table 2.** OITE 2023–2024 Benchmarking (GPT-5 vs. Gemini 2.5 Pro)

Category	GPT-5 accuracy (%)	Gemini 2.5 pro accuracy (%)	Resident benchmark (%)	Source
Overall (N=412)	76.0	81.1	74.2–75.3	10,21
Text-Only (N=218)	79.8	87.2	—	10
Image-Containing (N=194)	71.6	74.2	—	10
Spine Subspecialty (N=38)	73.7	84.2	—	10
Basic Science (N=48)	75.0	95.8	—	10
Trauma (N=58)	82.8	69.0	—	10
Sports Medicine (N=34)	94.1	88.2	—	10
Hand and Wrist (N=28)	42.9	57.1	—	10
GPT-5 dominant error mode	Faulty reasoning 52.5%	—	—	10
Gemini 2.5 Pro dominant error mode	—	Stem misinterpretation 43.6%	—	10

All model performance values derive from a single cross-sectional benchmarking study conducted in Vietnam, in which two large language models were evaluated in parallel on 412 Orthopaedic In-Training Examination-style questions from the 2023–2024 cycle.^10^ Abbreviations: GPT, generative pretrained transformer; N, number of questions; OITE, Orthopaedic In-Training Examination. Percentages represent question-level accuracy unless otherwise specified. Resident benchmark values represent reported resident performance ranges for comparison. Blank cells indicate that the metric was not applicable or not reported for that model or category. Hand and Wrist was the lowest-scoring category for both models.

### Administrative Automation and Coding

Administrative applications focused on documentation, coding, and extraction of billable procedural details. LLMs have been incorporated into electronic health record workflows as ambient documentation tools and operative-report drafting aids.^2^ In spine surgery coding, prompt-engineered LLMs extracted CPT codes from operative notes with an AUROC of 0.87 and AUPRC of 0.67.^5,22^ These findings support a potential role for LLMs in coding-assist workflows, particularly when operative reports contain the technical details needed to distinguish procedure types.

Coding applications also require close expert oversight. Spine-related CPT updates for fiscal year 2026 include sacroiliac joint fusion codes 27278 and 27279 and the annular closure device add-on code +63032.^23^ Because reimbursement depends on accurate code assignment, and because orthopaedic surgery faces estimated efficiency-adjustment reductions in the 2026 Medicare Physician Fee Schedule, LLM-generated coding outputs require surgeon or certified coder validation before operational use.^24^

## Discussion

This scoping review suggests that LLMs in spine surgery are advancing most rapidly in tasks that depend on structured text, guideline retrieval, patient communication, education, and administrative support. However, their performance remains less reliable when tasks require image interpretation, spatial reasoning, individualized procedural selection, or integration of subtle clinical modifiers. This mismatch supports a central “performance paradox”: LLMs may perform well on standardized examinations or constrained text-based prompts while remaining insufficiently dependable for autonomous spine-surgical decision-making.^5,7,10^

The clearest example is the gap between benchmark performance and real-world clinical synthesis. Gemini 2.5 Pro achieved high accuracy on OITE Basic Science items, but agreement with expert procedure selection in minimally invasive spine surgery triage was only fair at the case level and improved mainly when the task was simplified to binary surgical triage.^10,11^ This distinction is clinically important. Spine surgery often depends on features that are incompletely represented in text prompts, including radiographic morphology, prior surgery, multilevel disease, neurologic trajectory, sagittal alignment, patient goals, and surgeon-specific procedural thresholds.2^25^ As a result, LLMs may approximate broad triage decisions while failing to reproduce the finer judgments required for operative planning.^11^

Model errors also appear to reflect more than missing knowledge. GPT-5 errors on OITE questions were frequently attributed to faulty reasoning rather than simple factual deficits, indicating that larger or more current models may still generate confident but logically flawed conclusions.^10^ For clinical spine practice, this distinction matters because a plausible explanation can create a false sense of reliability. These findings support the use of LLMs as supervised adjuncts rather than independent clinical agents. In the near term, their safest role is likely to be clinician-mediated support for education, documentation, guideline summarization, patient-facing explanations, and preliminary triage rather than autonomous diagnosis or treatment selection.^5,7^

Technical approaches such as retrieval-augmented generation may partially address these limitations by grounding responses in curated, current, and specialty-specific sources. RAG-based systems can reduce knowledge lag and improve guideline alignment by allowing models to retrieve information from sources such as PubMed, society guidelines, or institutional protocols.^7^ For example, NotebookLM supplemented with NASS guidelines achieved high accuracy on guideline-based questions for degenerative spine conditions and outperformed baseline GPT-4o in that setting.^7,26^ These findings suggest that domain grounding may be more clinically useful than relying on general-purpose LLMs alone. This is particularly relevant in spine surgery, where recommendations, coding rules, payer policies, and procedural standards evolve over time.^7,27^

**Figure 3.**
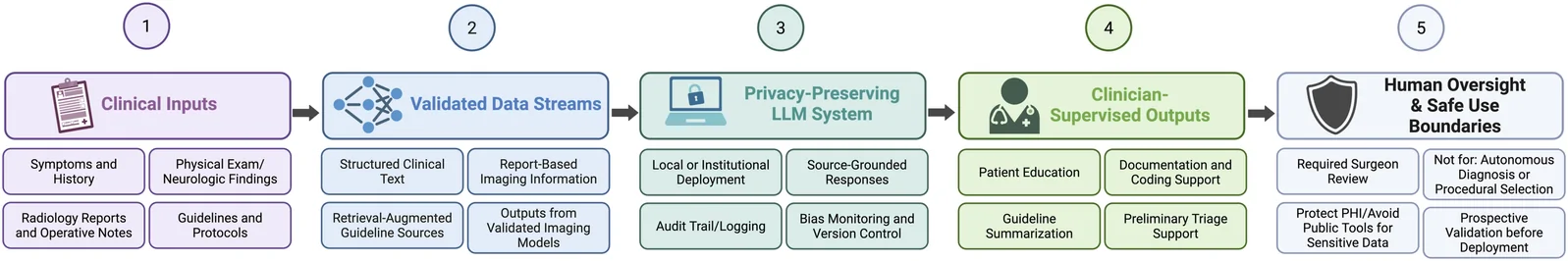
Clinical Implementation Pathway for Privacy-Preserving LLM Use in Spine Care. Proposed supervised workflow for integrating large language models into spine care. Clinical inputs are first routed through validated data streams, including structured text, imaging-derived information, retrieval-augmented guideline sources, and outputs from validated imaging models. These inputs then enter a privacy-preserving, source-grounded LLM system with institutional deployment, audit logging, bias monitoring, and version control. Model outputs are limited to clinician-supervised functions, including patient education, documentation and coding support, guideline summarization, and preliminary triage. Human oversight, protected health information safeguards, and prospective validation define the safe-use boundary and distinguish adjunctive support from autonomous diagnosis or procedural selection. Abbreviations: LLM, large language model; PHI, protected health information

Multimodal integration represents another important but still immature direction.^28^ Current general-purpose LLMs remain weak when asked to directly interpret imaging. GPT-4o performed poorly on trauma radiographs compared with emergency-medicine and orthopaedic specialists, underscoring the risk of using language models as stand-alone image interpreters.^29^ More realistic clinical architectures may combine dedicated imaging models for segmentation or classification with LLMs for report synthesis, guideline retrieval, and communication. In this model, the LLM does not replace specialized computer vision systems or clinician review but helps organize and explain information generated from validated inputs.^7^ A practical implementation pathway should therefore separate clinical inputs, validated data streams, privacy-preserving model deployment, clinician-supervised outputs, and explicit human oversight before any clinical use (Figure 3).

Implementation also requires attention to privacy, fairness, accountability, and workflow design. Spine care involves sensitive imaging, operative details, and longitudinal EHR data, making unrestricted use of third-party cloud-based tools inappropriate for protected clinical information.^7^ Local or institutionally governed models, privacy-preserving deployment, and clear data-use policies are likely prerequisites for clinical adoption. Bias is also a concern because LLM outputs may reflect inequities embedded in training data or clinical documentation patterns, potentially affecting treatment recommendations for underrepresented groups.^1,7^ Prospective fairness audits, diverse validation cohorts, and transparent reporting of model behavior across demographic and clinical subgroups will be necessary before these tools can be responsibly deployed.^7^

Legal accountability remains unresolved. If an LLM contributes to an erroneous recommendation, responsibility among the clinician, institution, vendor, and model developer is not yet clearly defined. No U.S. malpractice framework has been tailored specifically to LLM-mediated surgical decision-making, and current approaches rely on indirect analogies to existing medical-guidance precedent.^30^ This uncertainty reinforces the need for explicit human oversight, documentation of model use, institutional governance, and clear boundaries around permissible clinical functions. Table 3 summarizes the major implementation barriers and corresponding mitigation strategies for LLM integration in spine surgery.

**Table 3.** Summary of Clinical Integration Challenges and Recommendations

Challenge category	Key issue	Potential mitigation	Adoption readiness	Source
Reliability	Hallucinations/Reference Fabrications	RAG with authoritative source indexing	High (for text tasks)	7,26
Privacy	PHI risk in third-party clouds	Local/Open-source model deployment	Medium	7
Bias	Gender/Race inequities in data	Diverse dataset auditing and RAG	Low	1,7
Reasoning	Inability to handle low-prevalence modifiers	Multimodal fusion and expert oversight	Low	7,11
Readability	University-level default outputs	Plain language prompting and tailoring	High	7,18
Accountability	Black-box decision-making	Explainable AI (XAI) frameworks	Experimental	7,30

Abbreviations: AI, artificial intelligence; PHI, protected health information; RAG, retrieval-augmented generation; XAI, explainable artificial intelligence. Adoption readiness reflects the relative feasibility of near-term clinical implementation based on current evidence and technical maturity. High indicates potential suitability for constrained text-based workflows; medium indicates feasible use with substantial safeguards; low indicates that further validation, bias assessment, or multimodal development is needed; experimental indicates that the approach remains primarily investigational.

These findings can be organized into three tiers of translational readiness, corresponding to the readiness gradient shown in Figure 2 and the adoption-readiness column of Table 3. The first tier comprises applications suitable for near-term supervised implementation: radiology report simplification, patient-facing frequently asked questions, documentation support, source-grounded guideline retrieval, and coding assistance with mandatory human sign-off. These tasks are text-based, produce output that a clinician can verify in seconds, and carry no autonomous decision authority.^7,17-19,22^ The second tier comprises applications that require prospective validation before clinical use: spinal cord stimulation candidacy screening, binary surgical versus nonsurgical triage, emergency consultation support, and structured extraction of standardized scores such as the Spinal Instability Neoplastic Score and the Pfirrmann grade. Agreement in this tier ranges from fair for triage judgments to high for structured score extraction, but the supporting evidence is uniformly retrospective or simulation-based, with no linkage to patient outcomes.^7,11,12,14,16^ The third tier comprises applications that remain experimental: direct image interpretation, quantitative radiographic measurement such as Cobb angle determination, selection of a specific operative procedure, and individualized prognostic counseling. Performance in this tier falls outside accepted clinical tolerance, and no current evidence supports use beyond research settings.^11,15,18,29^ Stratifying the evidence in this way provides a practical basis for departmental decision-making, separating what may be adopted now under clinician supervision from what should be studied prospectively and what should be deferred.

## Conclusion

LLMs show meaningful promise in spine surgery, particularly for text-based education, patient communication, documentation, coding, guideline retrieval, and clinician-supervised triage.^31^ Their current limitations are most apparent in image-dependent assessment, individualized operative planning, and complex clinical reasoning, where benchmark performance does not reliably translate into safe decision-making. The next phase of development should emphasize specialty-grounded RAG systems, multimodal architectures that integrate validated imaging models, local privacy-preserving deployment, and prospective evaluation in real clinical workflows.^3,32^ Until these systems demonstrate reliability, fairness, and accountability in spine-specific settings, LLMs should be used as clinician-supervised adjuncts rather than autonomous diagnostic or therapeutic tools.

## Supplemental Material

Supplemental Material - Large Language Models in Spine Surgery: A Scoping Review of Clinical Efficacy, Technical Integration, and Ethical Paradigms Supplemental Material for Large Language Models in Spine Surgery: A Scoping Review of Clinical Efficacy, Technical Integration, and Ethical Paradigms by Samer G. Salman, Rohan A. Phadke, Anne E. Tatooles, Adithya Nair, Alireza Tavakkoli, and James Rizkalla in Global Spine Journal.
