# Supplementary material for: Large Language Models in Spine Surgery: A Scoping Review of Clinical Efficacy, Technical Integration, and Ethical Paradigms
Source: Global Spine J. 2026 Aug 20:21925682261481450. Online ahead of print. doi: 10.1177/21925682261481450 (PMC13493616; doi:10.1177/21925682261481450)
Supplement: Supplementary file 1 [file sj-pdf-1-gsj-10.1177_21925682261481450.pdf]

Supplementary Methods

Electronic Search Strategy for "Large Language Models in Spine Surgery: A Scoping Review of Clinical Efficacy, Technical Integration, and Ethical Paradigms"

Search strategy and information sources

A comprehensive literature search was designed for PubMed/MEDLINE, Embase, Scopus, Web of Science Core Collection, and the Cochrane Library. The search covered publications from January 1, 2023 through May 15, 2026, and was restricted to English-language records. The strategy combined two core concepts: (1) large language models and generative artificial intelligence, and (2) spine surgery, spinal disease, or spine-specific clinical care. To improve specificity while preserving sensitivity for the prespecified review scope, broad terms that commonly retrieve non-spine orthopedic or neurosurgical literature were not required in the final search. Likewise, task-specific terms such as patient education, coding, triage, or examination performance were not required because eligible applications could span multiple clinical and technical domains.

Search limits. English language; publication dates January 1, 2023 through May 15, 2026. Where supported by the database interface, publication-type filters were limited to peer-reviewed articles and reviews, with conference abstracts excluded.

Supplementary searching. Reference lists of included studies and relevant review articles were examined to identify additional potentially eligible reports. Records identified through citation searching were tracked separately from database-derived records for PRISMA-ScR reporting.

Overview of electronic databases

Database	Platform / field structure	Language / type limits
PubMed/MEDLINE	PubMed; title/abstract	English
Embase	Embase.com; title/abstract	English; exclude conference abstracts
Scopus	Title and abstract	English; Article or Review
Web of Science Core Collection	Title and abstract	English; Article or Review
Cochrane Library	Search Manager; title and abstract	English where applicable

Database-specific search strategies

1. PubMed/MEDLINE

Platform/fields: PubMed; title/abstract fields with date and language restrictions embedded in the query.

```
(
  "large language model"[tiab]
  OR "large language models"[tiab]
  OR "generative artificial intelligence"[tiab]
  OR "generative AI"[tiab]
  OR ChatGPT[tiab]
  OR "Chat GPT"[tiab]
  OR "GPT-3"[tiab]
  OR "GPT-3.5"[tiab]
  OR "GPT-4"[tiab]
  OR "GPT-4o"[tiab]
  OR "GPT-5"[tiab]
  OR Gemini[tiab]
  OR Bard[tiab]
  OR Claude[tiab]
  OR Llama[tiab]
  OR "Med-PaLM"[tiab]
  OR "Microsoft Copilot"[tiab]
)
AND
(
  "spine surgery"[tiab]
  OR "spinal surgery"[tiab]
  OR "spine care"[tiab]
  OR "spinal disease"[tiab]
  OR "spinal diseases"[tiab]
  OR "cervical spine"[tiab]
  OR "thoracic spine"[tiab]
  OR "lumbar spine"[tiab]
  OR thoracolumbar[tiab]
  OR "spinal fusion"[tiab]
  OR "lumbar fusion"[tiab]
  OR "cervical fusion"[tiab]
  OR "spinal cord stimulation"[tiab]
  OR scoliosis[tiab]
  OR "spinal stenosis"[tiab]
  OR "disc herniation"[tiab]
  OR "disk herniation"[tiab]
  OR "degenerative disc disease"[tiab]
  OR "degenerative disk disease"[tiab]
  OR "cervical myelopathy"[tiab]
  OR "lumbar radiculopathy"[tiab]
  OR "cauda equina"[tiab]
)
AND ("2023/01/01"[dp] : "2026/05/15"[dp])
AND english[lang]
```

2. Embase

Platform/fields: Embase.com; title and abstract fields.

Additional limits: Exclude conference abstracts using the database publication-type filter.

```
(
  'large language model'
  OR 'large language models'
  OR 'generative artificial intelligence'
  OR 'generative AI'
  OR ChatGPT
  OR 'Chat GPT'
  OR 'GPT-3'
  OR 'GPT-3.5'
  OR 'GPT-4'
  OR 'GPT-4o'
  OR 'GPT-5'
  OR Gemini
  OR Bard
  OR Claude
  OR Llama
  OR 'Med-PaLM'
  OR 'Microsoft Copilot'
):ti,ab
AND
(
  'spine surgery'
  OR 'spinal surgery'
  OR 'spine care'
  OR 'spinal disease'
  OR 'cervical spine'
  OR 'thoracic spine'
  OR 'lumbar spine'
  OR thoracolumbar
  OR 'spinal fusion'
  OR 'lumbar fusion'
  OR 'cervical fusion'
  OR 'spinal cord stimulation'
  OR scoliosis
  OR 'spinal stenosis'
  OR 'disc herniation'
  OR 'disk herniation'
  OR 'degenerative disc disease'
  OR 'degenerative disk disease'
  OR 'cervical myelopathy'
  OR 'lumbar radiculopathy'
  OR 'cauda equina'
):ti,ab
AND [2023-2026]/py
AND [english]/lim
```

3. Scopus

Platform/fields: Scopus; TITLE-ABS fields.

Additional limits: Language = English; document type = Article or Review.

```
TITLE-ABS(
  "large language model"
  OR "large language models"
  OR "generative artificial intelligence"
  OR "generative AI"
  OR ChatGPT
  OR "Chat GPT"
  OR "GPT-3"
  OR "GPT-3.5"
  OR "GPT-4"
  OR "GPT-4o"
  OR "GPT-5"
  OR Gemini
  OR Bard
  OR Claude
  OR Llama
  OR "Med-PaLM"
  OR "Microsoft Copilot"
)
AND
TITLE-ABS(
  "spine surgery"
  OR "spinal surgery"
  OR "spine care"
  OR "spinal disease"
  OR "cervical spine"
  OR "thoracic spine"
  OR "lumbar spine"
  OR thoracolumbar
  OR "spinal fusion"
  OR "lumbar fusion"
  OR "cervical fusion"
  OR "spinal cord stimulation"
  OR scoliosis
  OR "spinal stenosis"
  OR "disc herniation"
  OR "disk herniation"
  OR "degenerative disc disease"
  OR "degenerative disk disease"
  OR "cervical myelopathy"
  OR "lumbar radiculopathy"
  OR "cauda equina"
)
AND PUBYEAR > 2022
AND PUBYEAR < 2027
```

4. Web of Science Core Collection

Platform/fields: Web of Science Core Collection; title (TI) and abstract (AB) fields.

Additional limits: Language = English; document type = Article or Review.

```
(
  TI=(
    "large language model" OR "large language models" OR
    "generative artificial intelligence" OR "generative AI" OR
    ChatGPT OR "Chat GPT" OR "GPT-3" OR "GPT-3.5" OR "GPT-4" OR
    "GPT-4o" OR "GPT-5" OR Gemini OR Bard OR Claude OR Llama OR
    "Med-PaLM" OR "Microsoft Copilot"
  )
  OR
  AB=(
    "large language model" OR "large language models" OR
    "generative artificial intelligence" OR "generative AI" OR
    ChatGPT OR "Chat GPT" OR "GPT-3" OR "GPT-3.5" OR "GPT-4" OR
    "GPT-4o" OR "GPT-5" OR Gemini OR Bard OR Claude OR Llama OR
    "Med-PaLM" OR "Microsoft Copilot"
  )
)
AND
(
  TI=(
    "spine surgery" OR "spinal surgery" OR "spine care" OR
    "spinal disease" OR "cervical spine" OR "thoracic spine" OR
    "lumbar spine" OR thoracolumbar OR "spinal fusion" OR
    "spinal cord stimulation" OR scoliosis OR "spinal stenosis" OR
    "disc herniation" OR "degenerative disc disease" OR
    "cervical myelopathy" OR "cauda equina"
  )
  OR
  AB=(
    "spine surgery" OR "spinal surgery" OR "spine care" OR
    "spinal disease" OR "cervical spine" OR "thoracic spine" OR
    "lumbar spine" OR thoracolumbar OR "spinal fusion" OR
    "spinal cord stimulation" OR scoliosis OR "spinal stenosis" OR
    "disc herniation" OR "degenerative disc disease" OR
    "cervical myelopathy" OR "cauda equina"
  )
)
AND PY=(2023-2026)
```

5. Cochrane Library

Platform/fields: Cochrane Library Search Manager; title and abstract fields.

Additional limits: Restrict publication dates to January 1, 2023 through May 15, 2026.

```
#1 ("large language model" OR "large language models" OR "generative artificial intelligence" OR
"generative AI" OR ChatGPT OR "Chat GPT" OR "GPT-3" OR "GPT-3.5" OR "GPT-4" OR "GPT-4o"
OR "GPT-5" OR Gemini OR Bard OR Claude OR Llama OR "Med-PaLM" OR "Microsoft
Copilot"):ti,ab

#2 ("spine surgery" OR "spinal surgery" OR "spine care" OR "spinal disease" OR "cervical spine" OR
"thoracic spine" OR "lumbar spine" OR thoracolumbar OR "spinal fusion" OR "spinal cord stimulation"
OR scoliosis OR "spinal stenosis" OR "disc herniation" OR "degenerative disc disease" OR "cervical
myelopathy" OR "cauda equina"):ti,ab
```

#3 #1 AND #2

Date limit: Publication date from January 1, 2023 to May 15, 2026, applied using the Cochrane Library date filter.
Language limit: English, applied where supported by the Cochrane Library interface.
